# Investigating the use of titanium dioxide (TiO_2_) nanoparticles on the amount of protection against UV irradiation

**DOI:** 10.1038/s41598-023-37057-5

**Published:** 2023-06-16

**Authors:** Reza Ghamarpoor, Akram Fallah, Masoud Jamshidi

**Affiliations:** 1 School of Chemical, Petroleum and Gas Engineering, Iran University of Science and Technology (IUST), Tehran, Iran; 2 Constructional Polymers and Composites Research Laboratory, School of Chemical, Petroleum and Gas Engineering, Iran University of Science and Technology (IUST), Tehran, Iran; 3 Department of Chemical Technologies, Iranian Research Organization for Science and Technology (IROST), Tehran, Iran

**Keywords:** Chemistry, Materials science, Nanoscience and technology

## Abstract

In this study, three samples of commercial titanium dioxide nanoparticles (TiO_2_) in different sizes were used to investigate their effect on the formulation of sunscreen creams. The aim was to evaluate their role in the performance of sunscreens (i.e. SPF, UVAPF, and critical wavelength). Then the particle size of these samples was determined by photon correlation spectroscopy methods. As a result, the size of primary particles was reduced by using milling and homogenization methods at different times. The results showed that the particle size of samples TA, TB, and TC in the ultrasonic homogenizer decreased from 966.4, 2745.8, and 2471.6 nm to 142.6, 254.8, and 262.8 nm, respectively. These particles were used in the pristine formulation. Then the functional characteristics of each formulation were determined by standard methods. TA had the best dispersion in cream compared to other samples due to its smaller size (i.e. 142.6 nm). For each formulation, two important parameters, including pH and TiO_2_ dosage, were investigated in different states. The results showed that the formulations prepared with TA had the lowest viscosity compared to formulations containing TB and TC. SPSS 17 statistical software analysis of variance showed that the performance of SPF, UVAPF and λc in formulations containing TA had the highest levels. Also, the sample containing TAU with the lowest particle size values had the highest protection against UV rays (SPF). According to the photocatalytic functionality of TiO_2_, the photodegradation of methylene blue in the presence of each nanoparticle of TiO_2_ was studied. The results showed that smaller nanoparticles (i.e. TA) had more photocatalytic activity under UV–Vis irradiation during 4 h (TA (22%) > TB (16%) > TC (15%)). The results showed that titanium dioxide can be used as a suitable filter against all types of UVA and UVB rays.

## Introduction

**Figure 1:**
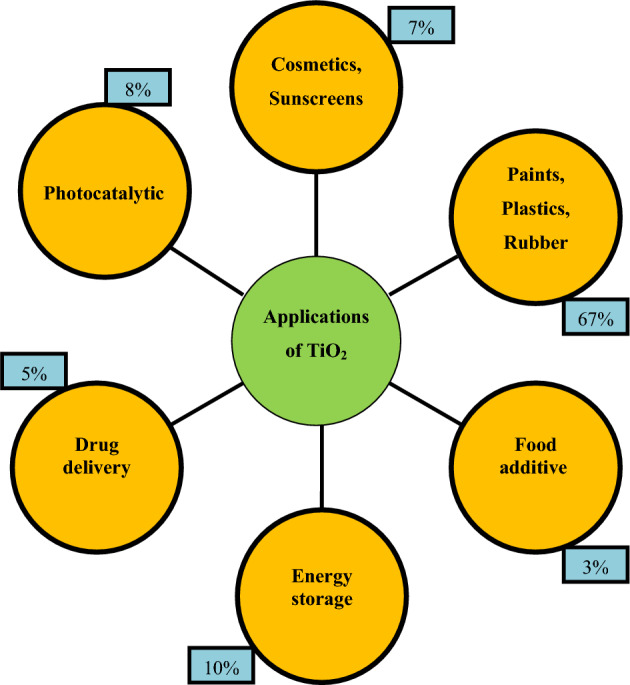
Main applications of TiO_2_ nanoparticles^13,14^.

Every year, significant amounts of titanium dioxide are used in cosmetic and pharmaceutical products, both as pigments and as sunscreen filters^1^. In recent years, along with the development of nanotechnology in the world and due to the unique characteristics of titanium dioxide nanoparticles and other nanoparticles, the use of the material in rubber^2^, photoelectrocatalyst^3^, airborne nanoparticle^4,5^, oil–water separation^6,7^, liposome productions^8^, enhanced oil recovery^9^, pharmaceutical, cosmetic and health industries, especially in sunscreens has increased significantly^10–12^. Figure 1 shows the main applications of TiO_2_ and their usage percentage in different industries^13,14^. Considering the increasing tendency to consume cosmetic and medical products, to use these products to maintain and improve the health of society, it is very important to control the safety and performance of these products^15^.

Nano titanium dioxide (TiO_2_) in spite of its mass state is transparent and is effective as a photocatalyst and an absorber of ultraviolet radiation^16^. Its transparency and absorbance features to ultraviolet radiation provide its effective use as a protective filter for sunscreen creams^17^. With the development of nanotechnology, pure or coated titanium dioxide nanoparticles have been used in the cosmetic and pharmaceutical industries^18^. Many studies on the safety of titanium dioxide nanoparticles have been conducted by various regulatory organizations, including the European Union, the US Food and Drug Administration, and the Australian Food and Drug Administration. On the other hand, by increasing the size of titanium dioxide particles, the ability to protect the skin against ultraviolet rays (especially UVB) decreased^19,20^.

Research has been conducted to achieve an optimal range of titanium dioxide particle sizes (i.e. between 50 and 150 nm) in sunscreen formulations, which not only minimizes safety concerns, but also results in the desired performance for the formulation^21,22^. To improve some properties of TiO_2_, most of them are covered with materials such as alumina or silica. These coatings can prevent some reactions between the surface of very active titanium dioxide nanoparticle crystals and the matrix in which they are spread. They can also lead to an easier distribution of titanium dioxide in the matrix^23,24^.

Titanium dioxide absorbs light at wavelengths of 275 to 405 nm and reflects light effectively due to its high refractive index^25^. Both of these effects, i.e. absorption and reflection, are effective in the power of protection obtained from titanium dioxide against ultraviolet rays^26^. According to theoretical calculations and data obtained from experimental studies, it has been proven that for TiO_2_ with particle size less than a micron, the amount of protection against rays UVB and UVB increase^2,27–29^.

Sunscreen creams use chemical properties (absorbing rays) or physical properties (blocking rays) to prevent UVB and UVA rays from penetrating the skin^30^. Their ability to protect against sunlight with a factor called sun protection factor (SPF) was investigated^31^. In sunscreen creams, in order to protect the skin against ultraviolet radiation, chemical or physical filters are used. Chemical filters mainly include organic materials with double and conjugated bonds, and physical filters are mainly metal oxides, including titanium dioxide and zinc oxide, which prevent radiation by reflecting or absorbing it^32,33^. The problem of chemical filters is the creation of free radicals that causes the reduction of their performance over time, while physical filters do not have this problem and have a longer and more effective performance^34^. The performance of sunscreen creams is measured by their ability to protect the skin against ultraviolet rays^35^. SPF is measured in two ways in the laboratory, i.e. in a simulated space with a living organism in the laboratory and the method by consuming the formulation directly on the human skin^36^.

In the standard method, the SPF factor is defined as the ratio between the energy required inducing the least response on human skin with and without the use of sunscreen products by using ultraviolet rays from an artificial source^37^. The term MED is the lowest amount of ultraviolet radiation that shows the first perceptible inflammation in the range of ultraviolet radiation, after 11 to 25 h of radiation. The MED value determined without the use of sunscreen cream indicated by MEDu and with the use of sunscreen cream by MEDp. The total average of individual SPFs (SPFi) for the product is defined as the ratio of the minimum protected erythema (MEDp) to the minimum unprotected erythema (MEDu)^38^. It should be noted that nanoparticles are toxic, that's why Torbati et al.^39^ investigated the toxicity of titanium nanoparticles in a study. The results showed that concentrations below 0.3 ppm were not toxic, but concentrations of 1 and 50 ppm were highly toxic.

In this research work, the use of titanium dioxide (TiO_2_) as a UV filter in the formulations of cosmetics and health products, especially sunscreens, has been investigated. Because titanium dioxide nanoparticles have a great tendency to aggregate and form coarse particles, as a result, experiments have been carried out to reduce these aggregates and turn them into smaller particles.

## Experimental section

### Titanium dioxide samples

Three commercial titanium dioxide samples available in the market in nano size were prepared and the particle size of these samples was determined. Due to the fact that in these samples the accumulation of particles had occurred, with the help of milling and homogenization methods, the size of the primary particles became smaller at different times and then was used in the reference formulations of sunscreen cream. Some features related to sunscreen performance, namely SPF, UVAPF and critical wavelength (λc) have been determined in these formulations. Also, the functional characteristics of each formulation in removing ultraviolet rays were determined. For each formulation, two critical parameters including pH and dosage of titanium dioxide in different states and the effect of these parameters on the size of the particles and the performance of the sunscreen formulation have been investigated. Three different samples of nano titanium dioxide, including: 1—uncoated, produced by DSM company, with primary particle size between 35 and 50 nm, 2—coated with PVP polymer material, produced by Indocom company, with primary particles size of 20 to 30 nm, 3—coated with polyisobutene, produced by LCW company, with the primary particles of 35 to 50 nm, have been used. These samples are coded with TA, TB and TC respectively.

### Preparation of formulations

The reference formulation with the least ingredients was used in order not to interfere with other unrelated factors in the experiment and only titanium dioxide was used as an ultraviolet filter. To prepare the formula, firstly, titanium dioxide at a concentration of 0.3 ppm (the concentration of nanoparticles was chosen due to its high toxicity according to reference^39^) was dispersed in oil (octyl-dodecanol) at a temperature of 80 °C. In a separate container, all the ingredients except water and glycerin were mixed together at a temperature of 74 °C. Then titanium dioxide dispersed in oil was added to this mixture and mixed completely. In the third container, water and glycerin were heated to 80 °C, and then the oil mixture was added to it and the mixture was completely uniform. Then the obtained suspension was slowly stirred until reaching a temperature between 35 and 40 °C. Table 1 summarizes some of the properties and usabilities of the ingredients in the sunscreen formulations. It is based on volume percentage except for titanium dioxide.

**Table 1:** Features and role of ingredients in sunscreen formulations.

Material’s name	Amount (volume %)	Performance
Titanium dioxide	0.3 ppm	Ultraviolet
Water	Until reaching the volume	Solvent
Cetyl alcohol	2–3%	Emulsifier and stabilizer
Glycerin stearate	3–4%	Emulsifier
Octyl dodecanole	8–12%	Solvent
Glycerin	7–15%	Solvent
Citric acid	Between 4 and 8 drops	pH tunning
Ammonium hydroxide	Between 4 and 8 drops	pH tunning

### Preparation of samples

Titanium dioxide samples were prepared in three ways and used in the sample. Firstly, an industrial homogenizer (manufactured by AICA company with the power of particles up to 300 nm), an ultrasonic homogenizer and a ball mill with zirconium balls were used. Industrial homogenizer uses mechanical force to agitate particles in a liquid while ultrasonic homogenizer benefits from ultrasound waves therefore. In all cases, titanium dioxide nanoparticles were dispersed in octyl-dodecanol oil. In order to volume measure titanium dioxide nanoparticles in the prepared formulations, first, 40 ml of water was added to 10 g of the sample and stirred well until the water phase was dissolved. Then 40 ml of ethyl ether was added to the mixture and mixed thoroughly. Then the mixture was poured into the decanter to separate organic phase from the aqueous phase. Subsequently, some experiments were performed on the organic phase.

### Photocatalytic activity of TiO_2_

The photocatalytic activity of three nanoscale particles of TiO_2_ was measured by monitoring photodecomposition of methylene blue (Purchased from Pars Company) in aqueous solution. For this means, the same amount of considered TiO_2_ nanoparticles e.g. (1) 142.6 nm, (2) 254.8 nm and (3) 362.8 nm, separately, was added to MB solution (100 ppm) in order to have 10 ppm solution of TiO_2_ and dispersed by ultrasound waves. The vessel was placed under UV–Visible lamp (high pressure Mercury lamp, 250 W) to irradiate light in the range of 300–600 nm (maximum absorption at 365 nm). Dye concentration was measured at 5-time intervals of 0, 30, 60, 120 and 240 min by UV–Vis spectrophotometer.

### Characterization

The determination of the size of titanium dioxide particles has been performed by photon correlation spectroscopy method and using the Master Sizer 2000 device made by Malvern company in England. The optometric device SPF 290 made by Optometrics of America was used for measuring functional parameters of formulations. To prepare the sample and as a substrate, 3 M Transpor surgical tapes made of polymethyl methacrylate were used. For this purpose, approximately 2 mg/cm^2^ samples were placed on the tape and set down in the machine. The scanning of the wavelengths has been adjusted with a distance of 2 nm and has been applied to the entire range of ultraviolet and visible rays. 12 scans were performed for each sample, and the results obtained for each product were the average of the results obtained from each scan. The ultraviolet light source used was a high pressure Mercury lamp, 250 W and a 140-W Xenon Arc Lamp. The morphology of the TiO_2_ nanoparticles was investigated by scanning electron microscope (SEM) (Hitachi). The optical characterizations for TiO_2_ samples were done using a UV–Vis diffuse reflectance spectrophotometer by a Shimadzu UV2550 spectrometer (Japan).

## Results and discussion

### Determination of particle size of titanium dioxide samples

Based on the measurements made by photon correlation radiometry, the particle size of the three studied titanium dioxide samples (TA, TB and TC) was determined and the results are summarized in Table 2.

**Table 2:** The particle size declared by the manufacturer and measured for titanium dioxide samples.

Sample code	Claimed on the label (nm)	Measured (nm)
TA	20–30	967
TB	35–50	2757
TC	30–50	2479

**Figure 2:**
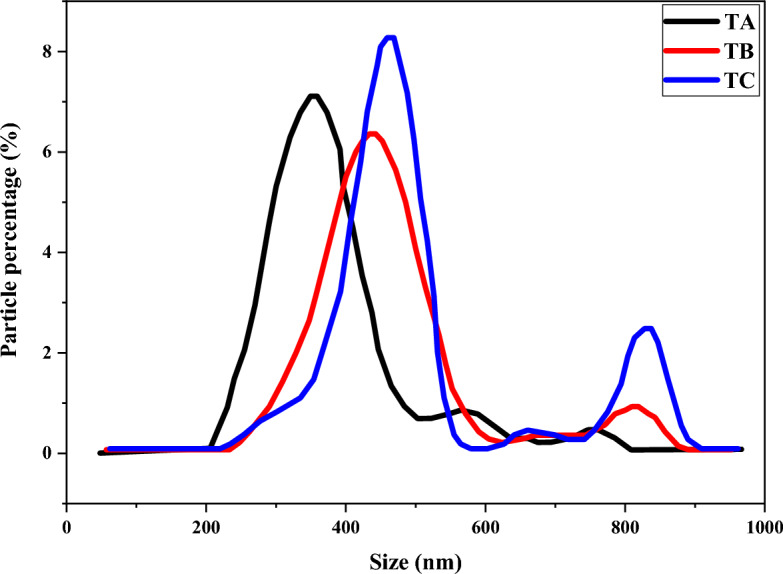
Particle size distribution diagram for TA, TB and TC processed samples.

**Figure 3:**
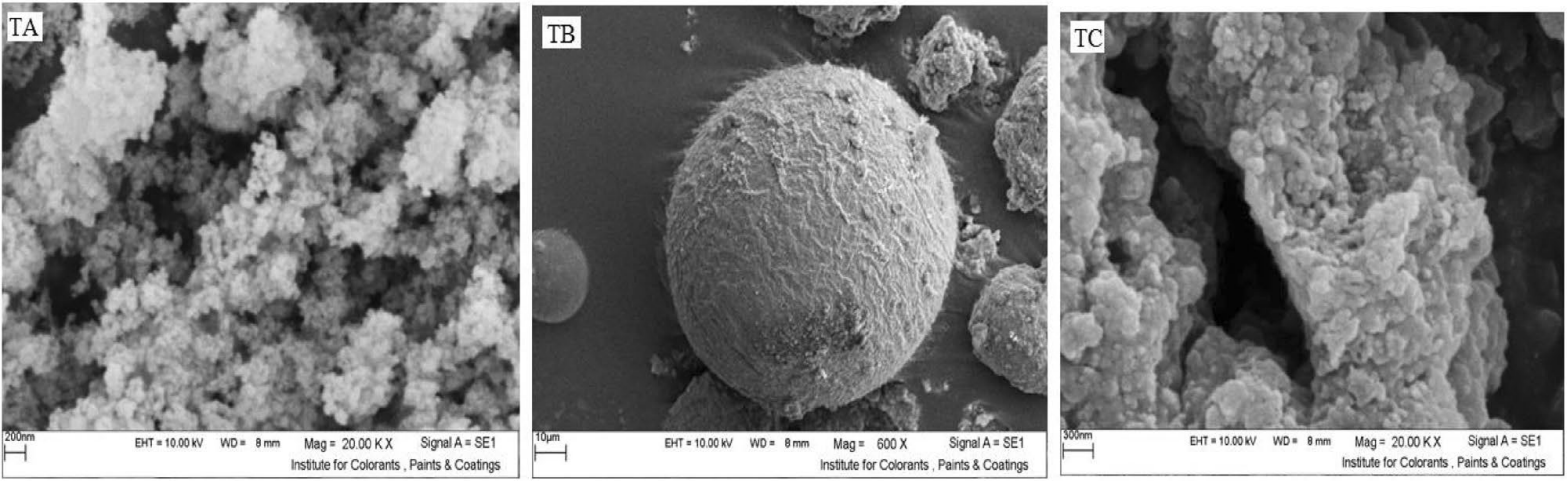
SEM images of TA, TB and TC samples.

As shown in Table 2, the results obtained from the average size of the particles were compared with what the manufacturer had announced, and due to the aggregation of the particles, there was a significant difference between the size declared on the label and the actual size of the particle. The graphs obtained from measuring the particle size of the samples by PCS are shown in Fig. 2 and their scanning electron microscope images are shown in Fig. 3.

According to the data in Table 2 and Figs. 2 and 3, all three samples have been subjected to agglomeration processes and their particle size has increased. TA sample showed the least and TB sample had the largest increase in particle size. Subsequently, some methods to eliminate the agglomeration and reduce the size of the particles have been applied before using the particles in the formulations.

### Reduction of particle size

Three different methods to reduce the size of particles, including 1—use of industrial homogenizer, 2—milling and 3—use of ultrasonic homogenizer for three samples of titanium dioxide have been investigated. For this purpose, each sample was tested four times and the average results are summarized in Table 3.

**Table 3:** The results obtained from reducing the particle size (nanometers) of titanium dioxide samples by various methods.

Sample code	TA			TB			TC		
Time (min)	Industrial homogenizer	Milling	Ultrasonic homogenizer	Industrial homogenizer	Milling	Ultrasonic homogenizer	Industrial homogenizer	Milling	Ultrasonic homogenizer
0	966.4	966.4	966.4	2745.8	2745.8	2745.8	2471.6	2471.6	2471.6
3	914.4	765.4	456.0	2498.6	2089.4	1742.0	2311.4	2227.4	1999.8
5	860.8	611.0	274.2	2288.2	1222.6	883.4	2226.4	1906.0	1440.0
10	830.6	545.2	148.2	2180.8	630.6	452.4	2112.8	1313.8	559.4
15	796.2	538.8	142.6	1974.6	629.6	254.8	1929.6	851.8	262.8

As the results of Table 3 show, the operation time for all three methods is considered up to 14 min, and among the three preparation methods, the use of ultrasonic homogenizer had the best performance in reducing the size of particles (142.6, 254.8, 362.8 nm respectively for TA, TB and TC in 15 min).

### Evaluating the characteristics of the reference formulation

For each type of titanium dioxide, five reference formulation samples have been prepared and evaluated, and the reference formulation samples are marked with the letter R at the end of their code. From the obtained 15 formulations, experiments were performed to determine viscosity and pH, and the obtained results are given in Table 4. For all five reference formulations prepared from each of the various titanium dioxide samples, the average pH and viscosity are summarized in Table 5.

**Table 4:** Results obtained from measuring pH and viscosity of 15 reference samples.

Formulation code	TiO_2_ sample	pH	Viscosity (MPa)
TA_R1_	TA	6.87	2135
TA_R2_	TA	6.73	2535
TA_R3_	TA	6.95	2148
TA_R4_	TA	6.84	2589
TA_R5_	TA	6.78	2778
TB_R1_	TB	7.02	3112
TB_R2_	TB	6.98	2600
TB_R3_	TB	7.12	2701
TB_R4_	TB	7.07	2574
TB_R5_	TB	6.93	2681
TC_R1_	TC	6.67	2213
TC_R2_	TC	6.75	2984
TC_R3_	TC	6.81	2504
TC_R4_	TC	6.62	2735
TC_R5_	TC	6.74	2318

**Table 5:** Average results of the specifications of the 3 reference formulations.

Formulation code	pH	Viscosity (mPas)
TA_R_	6.82	2435
TB_R_	7.02	2721
TC_R_	6.72	2554

### Studying the performance of formulations

For all three reference formulations prepared with three samples of titanium dioxide, their functional characteristics including SPF, UVAPF and critical wavelength (λc) were calculated and the results are presented in Table 6. It is worth mentioning that for each sample, UV scanning was performed five times and the average results are summarized in Table 6.

**Table 6:** The results obtained from the measurement of the performance characteristics of the reference formulations.

Formulation code	SPF	UVAPF	λ_C_
TA_R1_	8.38	6.25	383.9
TA_R2_	9.01	6.51	383.1
TA_R3_	8.41	6.42	384.2
TA_R4_	8.52	6.81	383.0
TA_R5_	8.75	6.41	384.9
TB_R1_	6.59	5.69	388.7
TB_R2_	6.14	5.56	388.8
TB_R3_	6.61	5.31	387.3
TB_R4_	6.61	5.58	387.0
TB_R5_	6.18	5.11	388.8
TC_R1_	7.57	6.16	385.4
TC_R2_	7.83	6.78	384.2
TC_R3_	6.93	6.01	385.9
TC_R4_	6.63	5.80	387.0
TC_R5_	7.46	6.64	384.7

One-way analysis of variance was calculated by SPSS 17 statistical software, in order to statistically investigate the effect of various reference formulations prepared from titanium dioxide on the three parameters SPF, UVAPF and λc. The results are shown in Tables 6 and 7.

**Table 7:** One-way analysis of variance for the comparison of three samples of titanium dioxide TC, TB and TA.

Formulation performance	Square average	Standard deviation	F	P
SPF	6.087	0.98	53.00	P < 0.001
	0.0115			
UVAPF	1.501	0.55	15.153	0.01
	0.099			
λ_C_	23.409	2.02	27.211	P < 0.001
	0.860			

**Figure 4:**
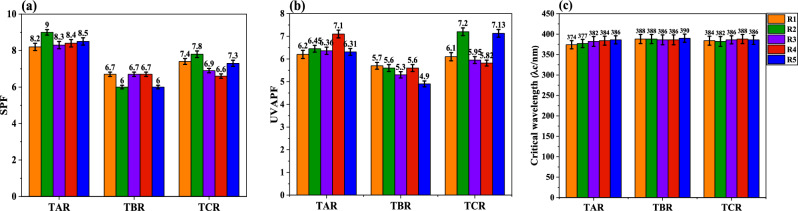
Statistical plots for three reference formulations, relative to performance for (**a**) SPF, (**b**) UVAPF and (**c**) critical wavelength.

According to Table 7, the P values were calculated less than 0.05, it proved that there was a significant difference between the groups. Table 8 shows the results of three groups’ follow-up analyses. Statistical graphs related to three performance characteristics for three types of titanium dioxide reference formulations are shown in Fig. 4.

**Table 8:** Results of follow-up analysis.

Performance	Formulation		Difference average	P	Confidence 95%	
					Low level	High level
SPF	TA_R_	TB_R_	2.18	< 0.001	1.59	2.78
		TC_R_	1.34	< 0.001	0.74	1.93
	TB_R_	TA_R_	−2.18	< 0.001	−2.78	−1.59
		TC_R_	−0.84	0.007	−1.24	−0.24
	TC_R_	TA_R_	−1.34	< 0.001	−1.93	−0.74
		TB_R_	0.84	0.007	0.24	1.44
UVAPF	TA_R_	TB_R_	1.02	0.001	0.47	1.58
		TC_R_	0.18	0.673	−0.37	0.74
	TB_R_	TA_R_	−1.02	0.001	−1.58	−0.47
		TC_R_	0.84	0.004	−1.30	−0.29
	TC_R_	TA_R_	−0.18	0.673	−0.73	0.47
		TB_R_	0.84	0.004	0.23	1.40
λ_C_	TA_R_	TB_R_	−4.28	< 0.001	−5.91	−2.63
		TC_R_	−1.61	0.053	−3.24	0.023
	TB_R_	TA_R_	4.28	< 0.001	2.63	5.91
		TC_R_	2.67	0.002	1.03	4.30
	TC_R_	TA_R_	1.61	0.053	0.023	3.24
		TB_R_	−2.67	0.002	−4.30	−1.03

As seen in the first row of Table 6 and Fig. 4a, TA_R_ formulation had a significant difference compared to the other two types of formulation, while there was no significant difference between the type of formulation and critical wavelength and UVAPF. The results were collected in Table 6 and Fig. 4b, c.

### The size of titanium dioxide particles in the formulation

By using various reference formulations (at the rate of 10%), the amount of accumulations and the behavior of various samples of titanium dioxide in these formulations have been investigated. In terms, the effect of particle size on the parameters related to the functional characteristics of the formulations Sunscreen includes SPF, UVAPF and λc measured. After these stages, two controllable parameters in the formulations, including pH and titanium dioxide dosages, were changed and their effects on the size of titanium dioxide particles and the aforementioned functional characteristics were measured again.

**Figure 5:**
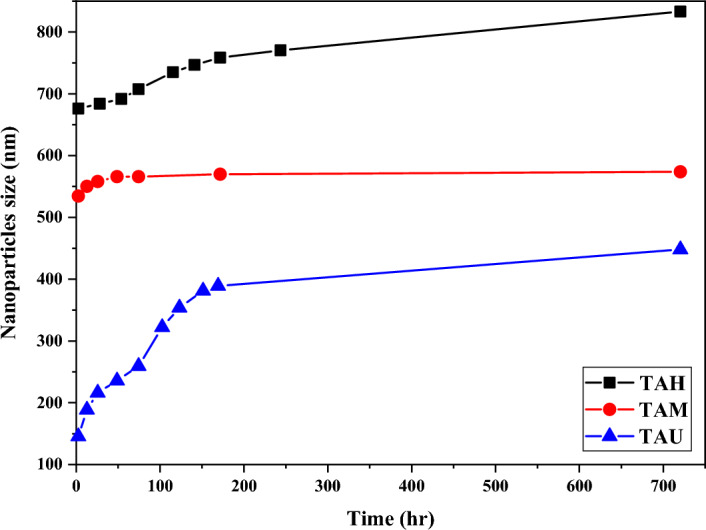
Studying the size of TA titanium dioxide particles, prepared by three different methods (industrial homogenizer H, mill M and ultrasonic homogenizer U) during a period of 1 month.

Among the three titanium dioxide samples, the TA sample, which is without modification or coating, has the lowest amount of aggregations and is easier to homogenize than the other samples. An easy solution to obtain a more suitable range of titanium dioxide nanoparticles is the rapid use of newly produced titanium dioxide or the pre-uniformization in small amounts by an ultrasonic homogenizer or a mill and the production of smaller volumes of the product. These solutions are also useful from an industrial and economic point of view. Figure 5 shows the changes in particle size for various samples of titanium dioxide prepared by different methods.

As shown in Fig. 5, it is clear that all three types of titanium dioxide samples have increased in particle size due to accumulation within one month. However, the intensity of increase in particle size for the sample prepared by ultrasonic homogenizer (TAU) had the highest increase rate among the three types of samples.

### Effect of particle size on SPF

**Figure 6:**
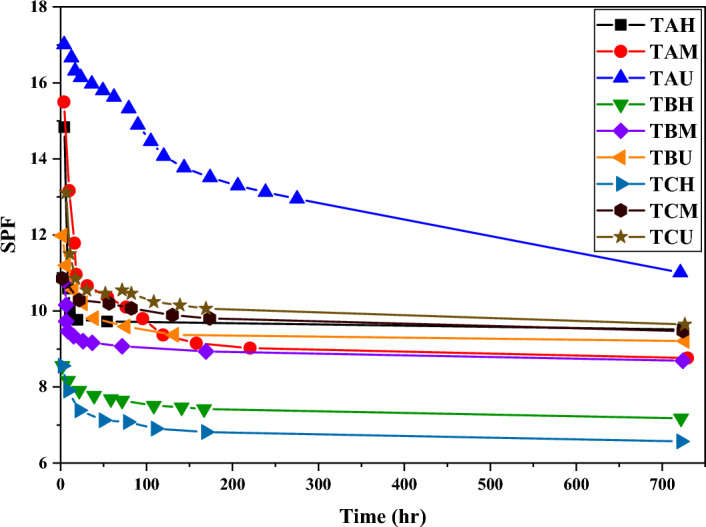
Investigating the change of SPF for various formulations with TA, TB and TC titanium dioxide samples during a period of 1 month.

In Fig. 6, the trend of SPF changes for three different titanium dioxide samples is compared. As it is clear from Fig. 6, there was a decrease in the SPF values of all formulations during the period of 1 month. However, among all these formulations, the sample containing TAU titanium dioxides with the smallest particle size created the highest SPF. It is generally known that the formulations with the lowest particle size values provided the highest amount of protection against UV rays (SPF) and it was known that high SPF values can be achieved with the help of titanium dioxide. Due to the re-accumulation of titanium dioxide after a period of more than one month, the SPF values obtained from different samples have been reduced to competitive values with a difference of 2 to 3 units.

### The effect of dosage on the size of titanium dioxide particles

**Figure 7:**
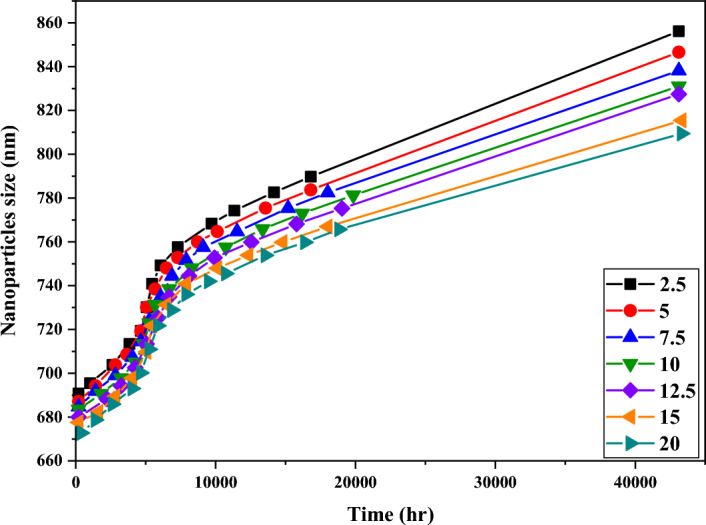
Investigating the effect of dosage on the particle size of TAH titanium dioxide sample.

The effect of dosage on the change of particle size in the formulation of sunscreen creams was studied and the results related to these measurements for the TAH sample are shown in Fig. 7. As seen in Fig. 7, the pattern of increasing the size of TAH samples was similar to each other. The remarkable thing was that the formulations with higher dosages of titanium dioxide had a lower particle size increase compared to samples with lower dosages of titanium dioxide. In the case of TBH and TCH samples, which had larger particle sizes than TAH samples, the particle size accumulation was independent of the dosage of titanium dioxide in the formulation. Of course, the coated surface of these two types of titanium dioxide (TBH and TCH samples) can also be a contributing factor to the accumulation of these samples.

Regarding all the samples prepared by the ultrasonic homogenizer, it can be seen that the increase in the size of the titanium dioxide particles with dosages of 10 and 20% has created an equal ratio and approximately the same sizes. While the amounts of accumulations in the samples with lower dosages (2.5%) had a greater increase in the size of the particles.

This can be related to the effect of PVP. In short, the higher the dosage of titanium dioxide in the formulation, the smaller the increase in particle size will be. However, the formulation used in this research had a relatively lower viscosity than the formulations available in the market, which causes more mobility of titanium dioxide particles, which resulted in an increase in the re-accumulation of titanium dioxide particles.

### Effect of dosage on SPF

**Figure 8:**
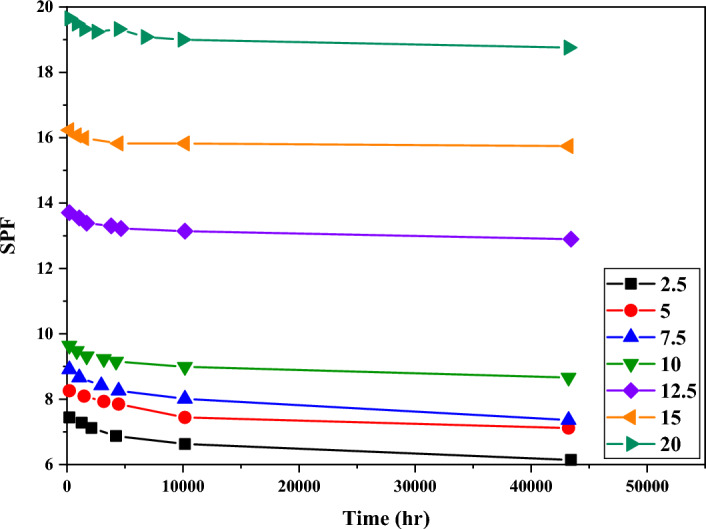
Investigating the effect of dosage on the SPF of TAH titanium dioxide sample particles.

As expected, with the increase in the dosage of titanium dioxide in the formulations, the SPF also increased strongly. The SPF values obtained with 20% dosage were about two times more than samples with 10% titanium dioxide. However, the difference between 2.5 and 10% titanium dioxide samples was far less than this value. As a result, by using other ultraviolet filters in the formulation, 2.5 to 10% amounts of titanium dioxide can be used in the formulation and provide the necessary efficiency for the formulation. Nevertheless, it should also be kept in mind that the accumulation of titanium dioxide in lower dosages was more, which caused the phenomenon of whitening and consumer dissatisfaction. Figure 8 shows the comparison of SPF values for formulations with different dosages for the TAH sample.

In general, similar results were obtained for other samples. In addition, the smaller the size of the particles, the higher SPF values were obtained. The remarkable thing about the samples with 20% titanium dioxide is the small reduction of SPF over time, which is due to the high dosage of titanium dioxide in the formulation, which covered the effect of the particle size and in practice caused the SPF to be relatively independent of particle size.

### Effect of pH on SPF and UVAPF

**Figure 9:**
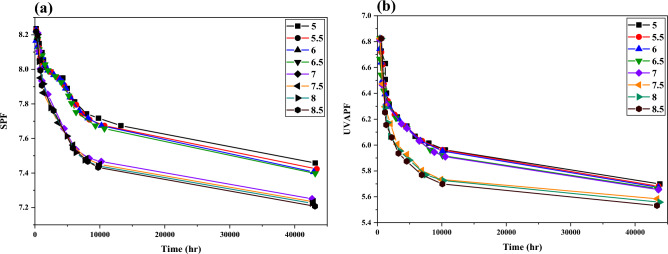
Investigating the change of (**a**) SPF with the change of pH in a period of 1 month for the TBH sample and (**b**) UVAPF with pH change in a period of 1 month for TCH sample.

When the pH was in the range of 5 to 8.5, the accumulation of particles increased and as seen before, this caused a decrease in SPF. Figure 9a shows these effects for TBH sample. This means that for formulations with different pH, the lowest SPF value obtained was between 5 and 8.5. Figure 9b shows the changes in UVAPF in the time periods of one month and with the change of pH for the sample of TCH titanium dioxides. As can be seen and predicted, the change of UVAPF also showed the same behavior as SPF, which was dependent on the size of the particles in the formulation. It can be seen that there was the smaller difference in UVAPF values at different pHs than the differences in SPF.

### Photocatalytic activity

**Figure 10:**
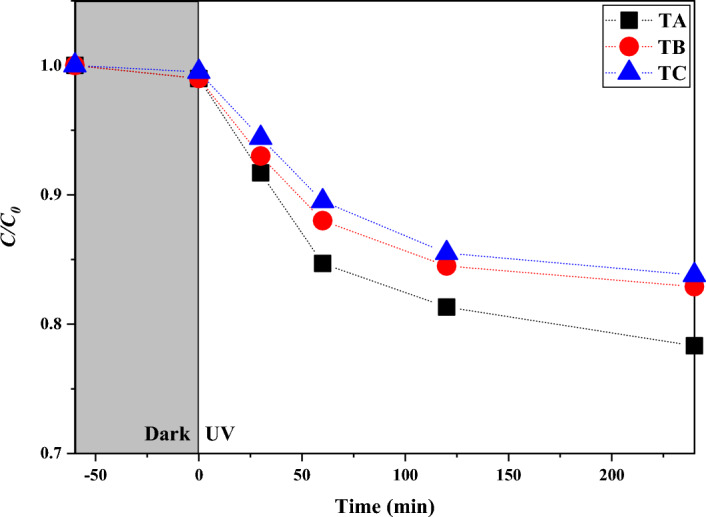
Photocatalytic degradation of MB with TiO_2_ samples.

Monitoring of decomposition rate of MB in aqueous solutions in the presence of three TiO_2_ nanoparticles (10 ppm), separately, was performed and the results are shown in the Fig. 10.

As the curves show in the Fig. 10, the best result was obtained in the presence of TiO_2_ with 142.6 nm size. Approximately 22% of MB was degraded in the presence of TiO_2_ (10 ppm) during 240 min under UV–Vis irradiation. Irradiation of UV–Vis light on the TiO_2_ surface resulted in movement of photoexcited electrons from conduction band (CB) to valence band (VB). Photoexcited species e.g. electrons and holes produce new active radicals namely Reactive Oxygen SApecies (ROS) which in turn degrade MB in aqueous solution. The results show that TiO_2_ with smaller nanoparticle size demonstrated more photocatalyic effect and also is able to capture more amount of UV light and play efficiently role of UV-blocker against preservation of skin. These mutual factors play important role for TiO_2_ usability as sunscreen cream. Generally, the UV-blocker role of TiO_2_ must be prodomonant. Therefore the SCCS published standard limitations of TiO_2_ amounts and particles size in the cosmetic products^40^.

## Conclusions

The purpose of this research was to investigate the size of titanium dioxide particles on the functional characteristics of sunscreen creams, in experimental conditions, due to the accumulation of particles, their size did not match with what was specified on the label of the samples. After reducing the size of the particles in order to eliminate accumulation, their effect on the functional characteristics of sunscreens was also investigated. As it is clear from the results, titanium dioxide can be used as a suitable filter against all types of UVA and UVB rays. It was seen that the smaller the size of the titanium dioxide particles, the better the UVA and UVB protection. According to the device used in the industry for the production of sunscreen products, most sunscreen formulations will have titanium dioxide particle sizes above 100 nm. This reduced concerns about the skin penetration of titanium dioxide. In order to achieve the maximum protection of the product against UVA and UVB rays, measures such as using other chemical or physical filters along with titanium dioxide in the formulation, applied homogenization until reaching the smallest particle size of titanium dioxide and using high dosages of titanium dioxide were applied. Using more than 10% of titanium dioxide was difficult due to the existing complications for the stability of the formulation. In the concentration range between 5 and 10% of titanium dioxide, there was no significant difference between SPF and UVA values, and depending on the price of other chemical and physical filters, alternative filters can be used to reach higher efficiency values. Considering that SPF and UVAPF values in the range of 6 to 7.5 were the lowest compared to other pH values, it was better for the formulation to have values outside this range. Due to the fact that the pH of the skin is mainly in the range of 4.5 to 6, the pH between 5 and 6 will be suitable for the sunscreen formulation.

## Data Availability

All data generated or analysed during this study are included in this published article.

## References

[1] Weir A (2012). Titanium dioxide nanoparticles in food and personal care products. Environ. Sci. Technol..

[2] Ghamarpoor R, Jamshidi M (2022). Synthesis of vinyl-based silica nanoparticles by sol–gel method and their influences on network microstructure and dynamic mechanical properties of nitrile rubber nanocomposites. Sci. Rep..

[3] Eftekharipour F, Jamshidi M, Ghamarpoor R (2023). Fabricating core-shell of silane modified nano ZnO; Effects on photocatalytic degradation of benzene in air using acrylic nanocomposite. Alex. Eng. J..

[4] Xiang M (2021). Airborne nanoparticle collection efficiency of a TEM grid-equipped sampling system. Aerosol Sci. Technol..

[5] Xiang M (2021). Uncertainty assessment for the airborne nanoparticle collection efficiency of a TEM grid-equipped sampling system by Monte-Carlo calculation. Adv. Powder Technol..

[6] Ghamarpoor R, Jamshidi M (2022). Preparation of superhydrophobic/superoleophilic nitrile rubber (NBR) nanocomposites contained silanized nano silica for efficient oil/water separation. Sep. Purif. Technol..

[7] Ghamarpoor, R. & Jamshidi, M. Silanizing nano SiO2 and its application in recycled nitrile rubber to prepare super oil resistant/superhydrophobic/superoleophilic oil/water separator. *J. Environ. Chem. Eng.* 107971 (2022).

[8] Bressot C (2016). Exposure assessment based recommendations to improve nanosafety at nanoliposome production sites. J. Nanomater..

[9] Ghamarpoor R, Ebrahimabadi A (2019). Optimum design of water-based drilling fluid in shale formations in Khangiran oilfields. Prog. Ind. Ecol. Int. J..

[10] Raj S (2012). Nanotechnology in cosmetics: Opportunities and challenges. J. Pharm. Bioallied Sci..

[11] Kaur IP, Agrawal R (2007). Nanotechnology: A new paradigm in cosmeceuticals. Recent Pat. Drug Deliv. Formul..

[12] Warheit DB (2018). Hazard and risk assessment strategies for nanoparticle exposures: How far have we come in the past 10 years?. F1000Research.

[13] Das, R., Ambardekar, V. & Bandyopadhyay, P.P. *Titanium Dioxide and Its Applications in Mechanical, Electrical, Optical, and Biomedical Fields.**Titanium Dioxide-Advances and Applications* (2021).

[14] Cox, A., Chandra, P. & Sharma, N. *Application of Titanium Dioxide Nanoparticles in Consumer Products Raises Human Health Concerns: Lessons from Murine Models of Toxicity.**Nanomaterial Biointeractions at the Cellular, Organismal and System Levels*. 3–51 (2021).

[15] Anderson P, Chisholm D, Fuhr DC (2009). Effectiveness and cost-effectiveness of policies and programmes to reduce the harm caused by alcohol. Lancet.

[16] Arora, I. *et al*. Advances in the strategies for enhancing the photocatalytic activity of TiO_2_: Conversion from UV-light active to visible-light active photocatalyst. *Inorgan. Chem. Commun.* 109700 (2022).

[17] Egambaram OP, Kesavan Pillai S, Ray SS (2020). Materials science challenges in skin UV protection: A review. Photochem. Photobiol..

[18] Jassal PS (2022). Green synthesis of titanium dioxide nanoparticles: Development and applications. J. Agric. Food Res..

[19] Newman MD, Stotland M, Ellis JI (2009). The safety of nanosized particles in titanium dioxide-and zinc oxide-based sunscreens. J. Am. Acad. Dermatol..

[20] Jovanović B (2015). Critical review of public health regulations of titanium dioxide, a human food additive. Integr. Environ. Assess. Manag..

[21] Tyner K (2011). The state of nano-sized titanium dioxide (TiO_2_) may affect sunscreen performance. Int. J. Cosmet. Sci..

[22] Chen MC (2022). Titanium dioxide and other nanomaterials based antimicrobial additives in functional paints and coatings. Prog. Org. Coat..

[23] Lee K, Mazare A, Schmuki P (2014). One-dimensional titanium dioxide nanomaterials: Nanotubes. Chem. Rev..

[24] Liao C, Li Y, Tjong SC (2020). Visible-light active titanium dioxide nanomaterials with bactericidal properties. Nanomaterials.

[25] Khorasaninejad M (2016). Visible wavelength planar metalenses based on titanium dioxide. IEEE J. Sel. Top. Quantum Electron..

[26] Saha S, Victorious A, Soleymani L (2021). Modulating the photoelectrochemical response of titanium dioxide (TiO_2_) photoelectrodes using gold (Au) nanoparticles excited at different wavelengths. Electrochim. Acta.

[27] Katangur P, Patra PK, Warner SB (2006). Nanostructured ultraviolet resistant polymer coatings. Polym. Degrad. Stab..

[28] Lavoie J, Boulay AM, Bulle C (2022). Aquatic micro-and nano-plastics in life cycle assessment: Development of an effect factor for the quantification of their physical impact on biota. J. Ind. Ecol..

[29] Ghamarpoor R, Jamshidi M (2023). Synergistic effect of microwave assisted devulcanization of waste NBR rubber and using superhydrophobic/superoleophilic silica nanoparticles on oil–water separation. Alex. Eng. J..

[30] Korać RR, Khambholja KM (2011). Potential of herbs in skin protection from ultraviolet radiation. Pharmacogn. Rev..

[31] Sharma T, Tyagi V, Bansal M (2020). Determination of sun protection factor of vegetable and fruit extracts using UV–Visible spectroscopy: A green approach. Sustain. Chem. Pharm..

[32] Pawlowski S (2021). EcoSun Pass: A tool to evaluate the ecofriendliness of UV filters used in sunscreen products. Int. J. Cosmet. Sci..

[33] Santander Ballestín S, Luesma Bartolomé MJ (2023). Toxicity of different chemical components in sun cream filters and their impact on human health: A review. Appl. Sci..

[34] Silva C, Biscaia EC (2003). Genetic algorithm development for multi-objective optimization of batch free-radical polymerization reactors. Comput. Chem. Eng..

[35] Bernstein EF (2020). Beyond sun protection factor: An approach to environmental protection with novel mineral coatings in a vehicle containing a blend of skincare ingredients. J. Cosmet. Dermatol..

[36] Quiñones R (2019). Comparing free radicals in sunscreen-treated pig skin by using electron paramagnetic resonance spectroscopy. J. Chem. Educ..

[37] Zou W (2022). Sunscreen testing: A critical perspective and future roadmap. TrAC Trends Anal. Chem..

[38] Azizi E (1987). A more reliable index of sunscreen protection, based on life table analysis of individual sun protection factors. Br. J. Dermatol..

[39] Torbati TV, Javanbakht V (2020). Fabrication of TiO_2_/Zn_2_TiO_4_/Ag nanocomposite for synergic effects of UV radiation protection and antibacterial activity in sunscreen. Colloids Surf. B.

[40] *SCCNFP, Opinion of the Scientific Committee on Cosmetic Products and Non-Food Products Intended for Consumers Concerning Titanium Dioxide*. (European Commission Brussels, 2000).

